# Recognizing species as a new focus of virus research

**DOI:** 10.1371/journal.ppat.1009318

**Published:** 2021-03-04

**Authors:** Alexander E. Gorbalenya, Stuart G. Siddell

**Affiliations:** 1 Department of Medical Microbiology, Leiden University Medical Center, Leiden, the Netherlands; 2 Faculty of Bioengineering and Bioinformatics, Lomonosov Moscow State University, Moscow, Russia; 3 School of Cellular and Molecular Medicine, University of Bristol, Bristol, United Kingdom; University of Colorado Denver, UNITED STATES

## Abstract

Species taxa are the units of taxonomy most suited to measure virus diversity, and they account for more than 70% of all virus taxa. Yet, as evidenced by the content of GenBank entries and illustrated by the recent literature on SARS-CoV-2, they are the most neglected taxa of virus research. To correct this disparity, we propose to make species taxa a first choice for communicating virus taxonomy in publications concerning viruses. We see it as a key step toward promoting research on diverse viruses, including pathogens, at this fundamental level of biology.

## From virus pathogens to species

Viruses were discovered as miniscule agents causing infectious diseases [1–3], and this link to pathogenicity, which still pervades virology, is often reflected in the matching names for viruses and their associated diseases. Even in more recent times, where genomic characterization has come to play a central role, viruses continue to be viewed as phylogenetic clusters of entities that are closely related to the originally identified pathogens, sometimes with a division into subgroups such as genotypes or serotypes. The major virus pathogens of humans, including influenza virus, human immunodeficiency virus (HIV), and hepatitis C virus (HCV) were defined in this pathogen-centrist way [4–6]. However, with the advent of metagenomic (and transcriptomic) studies, which drive current virus discovery and account for the ever-increasing share of known viruses (see, e.g., [7,8]), a pathogen-based nucleation approach is not possible. Instead, these studies identify a virus as a genomic sequence from random sampling of the respective natural diversity, and typically, associated with no phenotype. The uncertain relationship between viruses that were defined as pathogens and those identified through metagenomics can best be resolved within the framework of a virus taxonomy that is based on a concept of species. This short opinion article calls for species to be recognized as the primary subjects of virology, to parallel a practice common elsewhere in biology.

### Species in biology and virology

Species are the units of evolution, and surveys of the corresponding taxa are used as a census of the abundance of natural biodiversity [9]. Yet virology is the only discipline that has not yet fully embraced this basic tenet of biology. We believe this is largely due to a fundamental and unresolved question: to what degree does virus taxonomy equate its species taxa with the biological entities, also named species, that are studied throughout the rest of biology? The issues of species definition, demarcation, and meaning in biology are legendary [10–12], but the same questions, which are just as relevant to virology and the classification of viruses [13–19], are not widely discussed among virologists. We maintain this is because the study of viruses in the context of their species has not entered the mainstream of virus research.

This can be illustrated by 2 examples. First, in GenBank, which is a popular entry point for everybody interested in biological information [20], the genome sequences of cellular organisms are specified using the respective species name. In contrast, the genome sequences of viruses are typically specified in the context of their virus names. Virus species are not commonly defined in the taxonomy hierarchy that GenBank uses to describe the position of viruses, although all other taxonomic ranks are listed. Indeed, the delineation of virus species at NCBI can most readily be located at pages exclusively dedicated to taxonomy, sending a message that this rank is of concern only for those interested in virus taxonomy, rather than to all virologists [14].

The second example we present is the literature relating to severe acute respiratory syndrome coronavirus 2 (SARS-CoV-2), the cause of the coronavirus disease 2019 (COVID-19) pandemic [21–23]. Although this virus was identified only a year ago, the rapidly accumulating literature already exceeds many tens of thousands of papers: a scale of attention afforded to only a few other viruses. The virus was named SARS-CoV-2 due to its assignment into the preexisting species *Severe acute respiratory syndrome-related virus* by the *Coronaviridae* Study Group (CSG) [24]. The paper title and its Abstract include the species name, and the public release of the preprint on the bioRxiv server and the subsequent publication of the paper by *Nature Microbiology* were commented on by diverse media, including *Nature* and *Science*. Indeed, the name SARS-CoV-2 has been broadly accepted by the international community. Nevertheless, the readers of the SARS-CoV-2 literature will be hard-pressed to find a few mentions of the cognate species taxon of this virus (*Severe acute respiratory syndrome-related virus*) in these publications, regardless of whether or not the original CSG paper is acknowledged.

**Fig 1:**
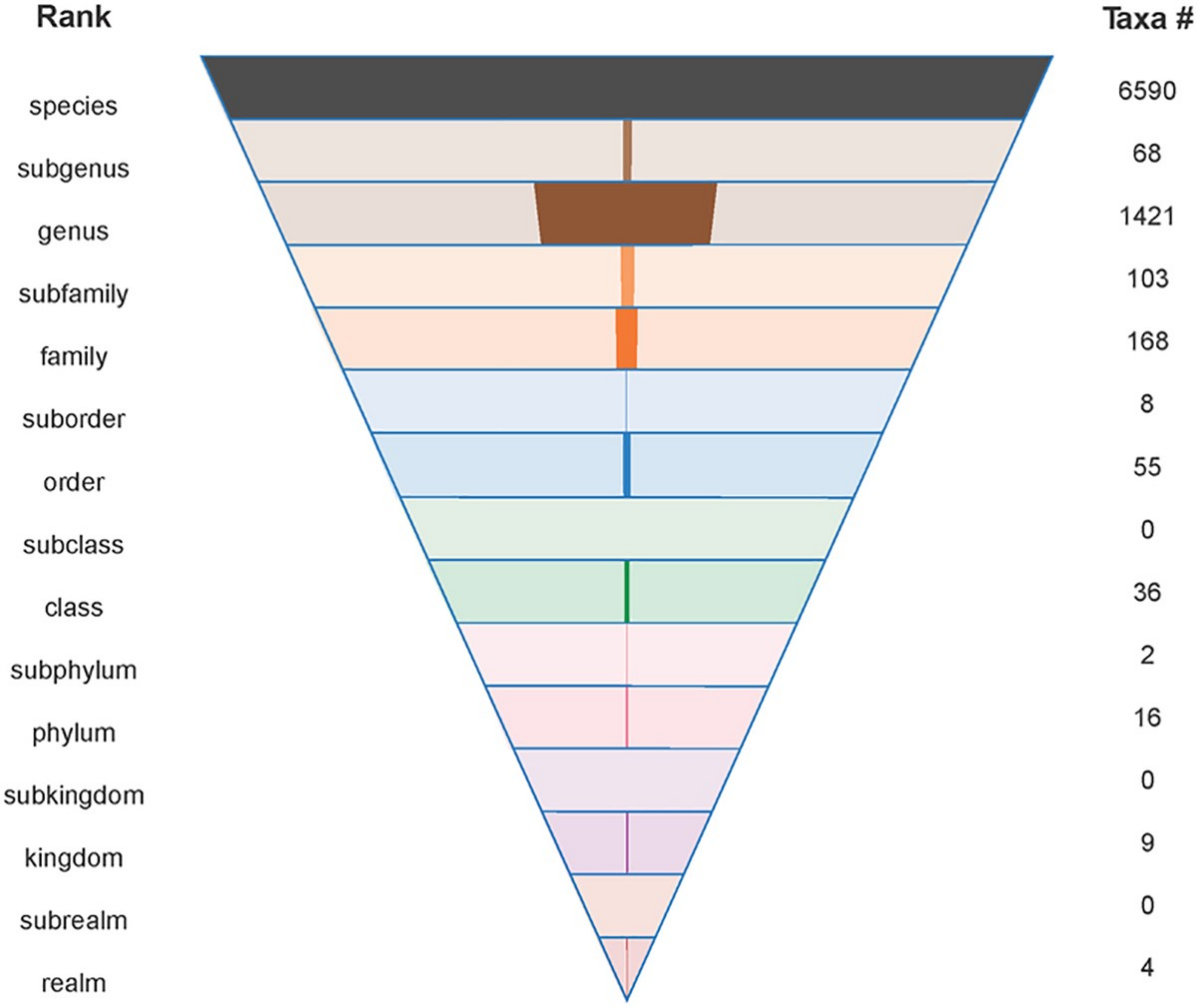
The 15 ranks of virus taxonomy are shown as an inverted pyramid, and the number of taxa currently assigned to each rank (December 2020) is shown both numerically and graphically.

One might argue that everybody knows where SARS-CoV-2 belongs, making an explicit reference to its multirank hierarchical taxonomy excessive in papers produced at the time when practical matters of containing the pandemic are the absolute priority. Indeed, many researchers chose not to refer to the coronavirus taxonomy in their publications. However, if any mention of taxonomy is made, the reference is typically to the family *Coronaviridae*, the genus *Betacoronavirus* or the subgenus *Sarbecovirus*. This disparity is especially notable since all of the aforementioned taxa were delineated and named at very different times. For example, the increasingly popular subgenus *Sarbecovirus* was first recognized only in 2018 [25], more than a decade later than the species *Severe acute respiratory syndrome-related virus* [26]. Given that virus species account for more than 70% of all virus taxa (**Fig 1**), this example reveals a disconnect between what virologists consider to be the most informative ranks of virus taxonomy (namely, any rank other than species) and what taxonomists think of as the cornerstone of virus taxonomy (the species rank). Essentially, it means that the development of virology proceeds largely without the benefit of the species concept, which is at the foundation of modern biology.

### Species in virology: The roots of neglect

So why do virologists not mention virus species in their work? There are probably many reasons, including (1) a reluctance to accept viruses as biological entities, despite extensive evidence to the contrary; (2) a predominant focus of virus research and its applications on circulating (often pathogenic) viruses that are typically a small subset of the species taxon, although they may be sufficiently divergent to be mistaken for a “species,” tacitly or explicitly; (3) a tradition in virology for citing taxa at the family and genus ranks, mainly because these ranks were established first [27]; and (4) the persistent confusion of species taxa names with those of the viruses that populate these taxa. For instance, HIV-1 and HIV-2 may be easily mistaken as acronyms of the species *Human immunodeficiency virus 1* and *Human immunodeficiency virus 2*, respectively. This is incorrect. The acronyms refer to the viruses, human immunodeficiency virus type 1 and human immunodeficiency virus type 2, and not to the species [5]. A similar opportunity for confusion may be observed in many other instances, for example, influenza A-D viruses, and, until recently, HCV, whose species was renamed to *Hepacivirus C* to address this problem [28]. It is to be hoped that the recent introduction of a Linnaean-like 15 rank structure into virus taxonomy [29], and the proposed introduction of a binomial species nomenclature for viruses [30], may contribute to improving the discrimination of virus names and species taxa names in a more consistent and transparent manner. However, this change will only have a real impact in virology and beyond if researchers recognize the importance of virus species and start referring to species taxa.

### Species in virology: Collaboration between virologists and the ICTV

Compared to the taxonomies of other biological entities, the development of virus taxonomy is uniquely supervised by a governing body, the International Committee on Taxonomy of Viruses (ICTV) [31]. This system provides a framework to develop a unified approach to virus classification and taxon nomenclature, which may be difficult to achieve otherwise, due to the unparalleled diversity of viruses and their ubiquitous infection of cellular organisms. However, this top-down arrangement, which has been applauded by some [32], might also create an impression that the development of virus taxonomy is relevant only to the ICTV. Nothing could be further from the truth. Virus taxonomy is a basis for organizing knowledge about known viruses within a hierarchical evolutionary-based framework, which, in turn, facilitates knowledge access, exploration, and generalization by the entire research community, as well as others who contribute to or benefit from advancements in virology.

This connection of taxonomy to virus research may not always be apparent, but it is easily seen if one looks at how the taxonomy of distinct virus families is developed in practice. This responsibility rests mainly with experts on the viruses belonging to different families, who join together to form specialized Study Groups (SG), such as the CSG mentioned above. There are currently more than 100 SGs, and the number is likely to increase dramatically in the next few years. Taxonomic proposals may be initiated by any researcher, but it is generally the SGs that delineate taxa using the methods and demarcation criteria believed, at the time, to be best for partitioning the natural diversity of the respective virus groups. This modular structure also allows for flexibility as the SGs are largely independent of each other and develop their remit as they see fit. These SGs are the main conduits to connect the ICTV to all who are concerned with viruses.

### Species in virology: Needs and benefits

We believe that to move the field forwards, the development of virus taxonomy has to be translated into research focusing on virus taxa, especially at the species level; a research area that is chronically underfunded, and how could it be otherwise? We are all aware of the chicken and egg mentality that bases research funding on prior research and creates a self-propagating circle of ignorance. What is needed is research that, for instance, includes the experimental verification of species by systematically probing the genetic compatibility of viruses at intra- and interspecies levels, as has been initiated for polioviruses and other viruses of the species *Enterovirus C* [33,34]. Another avenue to explore is the computational analysis of species demarcation, for example, the statistical evaluation of sequence discontinuity across different virus groups [35]. Only in this context will it become clear to virologists why, for example, HIV-1 and HIV-2 are assigned to 2 different species, while SARS-CoV and SARS-CoV-2 are 2 strains of a single species. Are these different assignments of a fundamental nature or the result of different approaches to species demarcation by the respective SGs? By resolving this question through empirical research on viruses at the species level, virologist will be able to understand better the relationship of taxonomy to biology and appreciate its implications, including the origins of pathogens. When studied in the context of their relatively long life span, the characterization of species can moderate the boom-and-bust cycles of interest and funding associated with virus pandemics, and advance virology in a conceptually coherent manner across the full spectrum of viruses.

### Concluding remarks: Focusing research on virus species

We call on all virologists, whether or not they are interested in taxonomy, to reflect on the importance of species taxa in their research and take the lead in referring to them in their publications and other communications. We hope that this will encourage others including, for example, GenBank to follow. Moreover, there should be more awareness among the editors of journals and science communications, as well as administrators of regulatory bodies, regarding the proper and consistent communication of virus species. In this regard, the ICTV has an important role to play in providing accurate and up-to-date information, which can be found in the current Master Species List and Virus Metadata Resource at the ICTV website (https://ictv.global/).

In our opinion, it would be logical to make species taxa, the principal unit of taxonomy, a first choice for communicating virus taxonomy in publications and other documents concerning viruses. This would represent a first but significant step forward in the effort to define a position for the virus entity in the natural world. Using species abundance as a census of virus diversity in different hosts (notably humans) [36] and biomes [37–40] would be another step in this direction. Importantly, these developments would also facilitate research into the full extent of viruses at the species level, as is common elsewhere in biology [41]. In practical terms, recognizing virus species as the principal subjects of virology will also expand the scale of the spatiotemporal framework that connects the studies of natural virus variation, cross-host transmission, and pathogenicity, and thus contribute to the understanding and control of virus infections as part of biome characterization and exploration of the biosphere.
